# Effects of complex enzyme preparation supplementation in a low-protein amino acid-balanced diet on production performance, serum biochemistry, nitrogen metabolism and nitrogen balance of mid-lactation dairy cows

**DOI:** 10.5713/ab.250890

**Published:** 2026-05-18

**Authors:** Caijuan Yue, Tianle He, Lisha Ma, Yapeng Qin, Yaqiong Ren, Rui Zhang, Meili Zhou, Qiao'e Zhang

**Affiliations:** 1 College of Animal Science and Technology, Ningxia University, Yinchuan, China; 2 Institute of Animal Science, Ningxia Academy of Agriculture and Forestry, Yinchuan, China; 3 Jiangzhang People's Government, Baoji, China

**Keywords:** Complex Enzyme Preparations, Dairy Cow, Low-protein Diet, Nitrogen Utilization Efficiency, Rumen-protected Amino Acids

## Abstract

**Objective:**

High-protein diets for lactating dairy cows reduce nitrogen (N) use efficiency (NUE) and cause environmental nitrogen pollution. Low-protein (LP), amino acid (AA)-balanced diets are a promising alternative but carry the risk of impairing nutrient digestibility and cow productivity. Exogenous enzyme preparations may mitigate these limitations, but their specific effects and underlying mechanisms in this feeding scenario remain not fully elucidated.

**Methods:**

Ninety mid-lactation Holstein dairy cows were assigned to 3 treatment groups in a randomized block design: (1) normal protein (NP) diet (17.30% crude protein, CP); (2) low-protein (LP) AA-balanced diet (16.09% CP, supplemented with rumen-protected lysine [RP-Lys] and rumen-protected methionine [RP-Met]); (3) LP diet plus 20 g/head per day of complex enzyme preparation (LPEP group). The 75 -day trial consisted of a 15 -day adaptation period and a 60 -day formal feeding period.

**Results:**

Compared with the NP and LP groups, the LPEP group showed significantly better production performance, apparent nutrient digestibility, feed conversion efficiency and milk quality (p<0.05). Serologically, LPEP cows had significantly higher serum albumin, lower serum urea nitrogen and blood glucose, and optimized AA profiles in both serum and milk. In the rumen, the LPEP diet significantly reduced ammonia nitrogen (NH_3_-N) concentration, while increasing microbial crude protein (MCP), acetic acid, propionic acid and total volatile fatty acids (TVFA), with no alteration of the overall ruminal fermentation pattern. Notably, the combination of LP AA-balanced diet and complex enzymes effectively reduced N excretion and improved NUE and N balance in dairy cows.

**Conclusion:**

Supplementation of complex enzyme preparation to a LP AA-balanced diet exerts a marked synergistic effect with rumen-protected AAs. This combined strategy simultaneously improves production performance, milk quality and NUE while reducing N waste, providing critical support for developing environmentally sustainable and efficient dairy feeding regimens.

## INTRODUCTION

The dairy industry, a core component of global animal agriculture, relies on lactating cows as its primary productive units, which have stringent nutritional requirements [1]. With the ongoing intensification of dairy production, the adoption of high-protein diets to maximize milk yield has become increasingly prevalent [2]. However, the long-term implementation of such diets not only increases feeding costs due to the high price of protein feeds but also results in excessive nitrogen (N) excretion [3,4]. Studies have demonstrated that under traditional high-protein feeding regimens, the nitrogen use efficiency (NUE) of lactating dairy cows typically ranges between 25% and 35%, with the upper limit of this range representing the optimal target achievable through precision nutrition strategies. A large proportion of unused N is therefore excreted from the body via feces and urine [2,5,6], leading to significant soil and water pollution. This results in significant pollution of soil and water bodies, and thus conflicting with the globally advocated principles of green animal husbandry [7,8]. Consequently, there is an urgent need to explore nutritional management strategies that simultaneously maintain production efficiency and environmental sustainability.

Within this context, the optimization of low-protein diets (LPD) has emerged as a prominent research focus in dairy cattle nutrition [9–11]. Targeted supplementation with rumen-protected essential amino acids (RP-EAAs) provides a strategy to reduce dietary protein content while optimizing the amino acid (AA) profile, thereby maintaining milk production performance, lowering feed costs, and reducing N emissions at the source [12,13]. However, simply reducing dietary protein levels without such adjustments often results in essential amino acid (EAA) deficiencies, which in turn can lead to declines in milk yield and milk protein content, as well as metabolic disorders [9]. Therefore, the pivotal challenge for the practical application of LPD lies in achieving a well-balanced dietary AA profile. This goal can be accomplished by supplementing rumen-protected EAAs (lysine [Lys], methionine [Met], and histidine [His]) to optimize the dietary AA profile and thereby meet the nutritional requirements of lactating dairy cows [3,12,14]. Existing research has confirmed that a low-protein (LP), AA-balanced diet can maintain production performance while reducing fecal and urinary N excretion [3,15]. Nevertheless, this nutritional strategy is often associated with compromised feed digestibility and suboptimal ruminal fermentation efficiency, which limits further improvements in NUE and creates a pressing need for novel feed additives to optimize the system [3,12].

Complex enzyme preparations, recognized as green feed additives, are widely used in animal production due to their efficacy in enhancing feed digestibility and regulating gut health [16]. In ruminant nutrition research, complex enzyme preparations containing non-starch polysaccharidases such as xylanase and cellulase can degrade structural carbohydrates in the plant cell wall, thereby disrupting the physical barriers that limit microbial access to intracellular nutrients [16,17]. This process enhances the release of nutrients and provides additional carbon sources for ruminal microbial growth [17,18]. Furthermore, complex enzyme preparations composed of fibrolytic and amylolytic enzymes can regulate ruminal microbial community composition and promote microbial protein (MCP) synthesis, thereby improving dietary NUE [17]. Theoretically, supplementation with complex enzyme preparations consisting of fibrolytic and amylolytic enzymes in LP, AA-balanced diets can enhance the digestibility of fibrous substances, thereby supplying more energy to ruminal microorganisms. This would compensate for potential deficiencies in ammonia nitrogen (NH_3_-N) resulting from protein reduction, stabilize or even increase MCP synthesis efficiency, and achieve a synergistic effect that combines “augmenting supply” with “reducing waste” [12,17]. However, existing studies on complex enzyme preparations have mainly focused on traditional protein diets, whereas reports regarding their effects on production performance and NUE in lactating dairy cows fed LP, AA-balanced diets remain relatively limited. It is therefore necessary to thoroughly investigate whether complex enzyme preparations can further improve production performance and N metabolism under AA-balanced conditions, and to elucidate their regulatory effects on relevant serum biochemical parameters associated with nutrition and metabolism.

Based on this rationale, the present study comprehensively evaluated the effects of supplementing a complex enzyme preparations to a LP, AA-balanced diet on lactating dairy cows. Through a combination of a feeding trial, a digestibility and metabolism trial, and analyses of blood parameters, milk composition, and ruminal fermentation parameters, we assessed production performance, apparent digestibility, serum biochemical indices, targeted metabolism of plasma and milk AA, N metabolism, and N balance. This study aims to elucidate the mechanisms of action and synergistic effects of the complex enzyme preparations within a LPD system. The findings are intended to provide a theoretical foundation and technical support for developing efficient, cost-effective, and environmentally friendly dietary formulations for lactating dairy cows.

## MATERIALS AND METHODS

### Animals, diets, experimental design, and feeding management

This study involved 90 mid-lactation Holstein cows with a body weight of 675±37.65 kg, mean lactation period of 93.55±6.42 days, parity of 2.43±0.08, milk production of 36.52±0.12 kg/d, and body condition score (BCS) of 2.90± 0.10. Based on milk yield, all cows were allocated using a randomized block design into three dietary treatment groups: a normal protein (NP) diet (NP group, 17.30% crude protein [CP]), a LP AA-balanced diet (LP group, 16.09% CP), supplemented with rumen-protected lysine (RP-Lys) and (RP-Met), and the LP group diet supplemented with 20 g/head/day of a complex enzyme preparations (LPEP group), with 30 cows in each group. Both the LPEP and LP groups were supplemented with 20 g/head/day of RP-Lys and 30 g/head/day of RP-Met on the basis of a LPD. The LPEP group was further supplemented with an additional 20 g/head/day of complex enzyme preparations incorporated into the dietary ration. The enzyme formulation used in this research is a cow-specific composite enzyme (SFC-061) provided by Cangzhou Xiasheng Enzyme Biotechnology, with main enzymatic activities including cellulase (activity≥1,600 U/g), xylanase (activity≥11,200 U/g), β-glucanase (activity≥3,000 U/g), neutral protease (activity≥ 500 U/g), and β-mannase (activity≥30 U/g). To distinguish the treatment methods for these dairy cows, we divided a pen capable of accommodating 220 cows (350 m in length) into three equal sections, each measuring 116–117 m in length, separated by manually movable steel pipes. Each cow was provided with an individual feeding box (90 cm×90 cm×90 cm) to facilitate the measurement of daily feed intake and leftover amounts (The feed trough is equipped with an automatic feeding door system [American Calan], and correspondingly, each cow wears a collar-type sensor). All diets were formulated based on the National Research Council (NRC 2001) to meet or exceed the nutritional requirements of mid-lactation Holstein cows. The diet formulations and nutritional values for the cows in this study are shown in Table 1. The added rumen-protected amino acid (RP-AA) and complex enzyme preparations were first mixed with the premix, then blended with the concentrate, and finally mixed with the total mixed ration (TMR) in a TMR mixer for dairy cow consumption. To ensure the uniform distribution of these RP-EAAs and complex enzyme preparations in the feed, the proportion of premix was increased from 2.50% in the NP diet to 4.00% in the LP and LPEP diets. The increased amount of premix served as a carrier. The premix formula was adjusted accordingly to maintain consistent final concentrations of vitamins, minerals, and other micronutrients in all treatment groups on a total diet basis. Feed waste was controlled within 10%, and dairy cows had free access to drinking water throughout the day. During the entire experimental period, all cows were fed three times a day (7:00; 14:00; 22:30) and milked three times a day (6:00; 14:00; 22:00). The experiment officially commenced after a 15-day pre-feeding period and lasted for 60 days.

### Feed sampling and chemical analyses

DMI was calculated based on the actual TMR weight offered to each cow and the corresponding absolute dry matter (DM) content. Fresh TMR rations added to the feed trough were collected weekly at a fixed time via multi-point sampling, samples were mixed thoroughly, and then obtained approximately 250 g using quadrat sampling as the final composite sample. Concurrently, at the time of TMR sampling, samples of forage and concentrate ingredients used in TMR preparation were also collected. All collected feed samples were temporarily stored at −20°C. Subsequently, feed samples were dried at 65°C for 48 h to determine DM content. The dried TMR samples were then ground through a 1-mm screen using a Cyclotec 1093 mill (Tecator AB, Höganäs) for subsequent analysis. To ascertain the absolute DM content, the feed samples underwent further drying at 105°C for 4 h. The compositional analysis of the TMR was performed according to established methods: DM, CP (calculated as Kjeldahl N×6.25), ether extract (EE), calcium, and phosphorus were determined using AOAC methods 930.15, 981.10, 920.39, 927.02, and 965.17, respectively [19]. Neutral detergent fiber (NDF) was analyzed according to Van Soest et al. [20] with heat-stable α-amylase and sodium sulfite. Acid detergent fiber (ADF) was determined following AOAC [19] method 973.18. Subsequently, the 72% sulfuric acid hydrolysis method was employed for the determination of lignin content [20]. All nutritional components were expressed on an ash-inclusive basis [21]. Starch content was quantified using a Total Starch Assay Kit (K-TSTA; Megazyme) and the RST-NaOH method.

### Milk sample collection and analysis

A milking machine equipped with a sampler was used to record the daily milk yield of the cows (ALPRO by DeLaval), with the total daily milk production calculated by summing the readings from the flow meters taken in the morning, afternoon, and evening. On the last day of each month during the experiment, 50 mL of milk sample was collected from each dairy cow per sampling event, with milk from the three daily milkings (morning, midday, and evening) mixed at a volume ratio of 4:3:3. One sample was placed in a preservative container containing bromophenol, refrigerated at 4°C, and sent to the Ningxia Academy of Agriculture and Forestry Sciences for milk composition analysis. The second sample was frozen at −20°C for subsequent determination of AA levels in milk. The concentrations of AAs in milk were determined using an automated AA analyzer (1290 Infinity II; Agilent Technologies), following the procedure described by Pereira et al. [22]. In addition, Energy corrected milk (ECM) = 0.327×milk yield (kg/d)+12.95×milk fat yield (kg/d)+7.65×milk protein yield (kg/d), 4% Fat corrected milk (4% FCM) = 0.4×milk yield (kg/d)+15×milk fat yield (kg/d).

### Blood sample collection and analysis

On the last day of the trial, blood was sampled from the coccygeal vein of each cow 2 h post-morning feeding. The blood was then dispensed into plain tubes and EDTA tubes separately for subsequent analyses. The blood without anticoagulants was allowed to rest for 30 mins before being centrifuged at 3,500×g for 15 mins. The serum was then separated into 2 mL centrifuge tubes and stored at −80°C for the determination of serum biochemical markers (Jiancheng Bioengineering Research Institute). Blood containing anticoagulants needed to be centrifuged immediately at 3,500×g for 15 mins and the plasma separated. Plasma AA concentrations were determined using an automatic AA analyzer (model 1290 Infinity II; Agilent Technologies), with the operational procedure referring to the description by Pereira et al. [22].

### Rumen fluid sample collection and analysis

On the last day of the trial, rumen fluid was collected 2 h post morning feeding using an oral collection tube (2 mm thick, 6 mm inner diameter; Anscitech) paired with a 150 mL syringe. The first 50–100 mL of rumen fluid, potentially contaminated with saliva, was discarded. Approximately 50 mL of rumen fluid was then re-extracted, and its pH was measured using a pH meter (PHSJ-3F; Shanghai Yifen Scientific Instrument). The rumen fluid was filtered through four layers of coarse cotton cloth and aliquoted into 15 mL and 2 mL cryotubes. The 15 mL cryotubes were stored at −20°C for total volatile fatty acids (TVFA) analysis, while the 2 mL cryotubes were snap-frozen in liquid N and stored at −80°C for NH_3_-N and MCP determination. The determination of VFA concentrations in rumen fluid was conducted following the method described by Li et al. [23]. The concentration of NH_3_-N in rumen fluid was measured via the phenol-hypochlorite assay according to the method of Hristov et al. [24]. Briefly, rumen fluid was centrifuged at 3,000×g for 10 min, and the supernatant was mixed with 20% trichloroacetic acid to precipitate proteins. After re-centrifugation at 10,000×g for 15 mins, the supernatant was incubated with phenol reagent and sodium hypochlorite reagent at 37°C for 20 min. The absorbance was measured at a wavelength of 550 nm, and quantification was performed using a standard curve generated with ammonium sulfate standard solutions. The concentration of MCP in rumen fluid was determined using the method described by Makkar and Becker [25]. MCP content was calculated using the formula:


(1) MCP (g/kg DM)=(Purine content×6.25×0.83)/0.116,

where 6.25 is the classic conversion factor for converting N content to protein content, 0.83 represents the proportion of total N in microbial cells relative to MCP, and 0.116 denotes the proportion of purine N in microbial cells relative to total N.

### Fecal and urine sampling and analysis

During the final three days of the study, fecal and urine samples were collected via spot sampling at scheduled times: 6:00, 12:00, and 18:00 on day one; 1:00, 7:00, and 14:00 on day two; and 10:00 and 21:00 on day three [26]. From each treatment group, 20 cows with homogeneous lactation performance, healthy physiological status and consistent basic traits (close to the group average) were selected by continuous recording and screening. Fecal samples were obtained from these cows through rectal palpation, immediately treated with 10% dilute H_2_SO_4_ (V/V), and stored at 4°C. Samples from the three days were pooled in equal ratios (1:1:1) to yield approximately 500 g of mixed feces, which was subsequently dried at 65°C to constant weight and ground to a particle size of 1 mm.

To determine absolute DM content, a sub-sample of the feed and feces was additionally dried at 105°C for 4 h. The contents of DM, CP, NDF, ADF, and EE in the feces were subsequently analyzed according to the same standard procedures for feed samples described above.

Acid-insoluble ash (AIA) was used as the endogenous marker to determine the apparent digestibility of nutrients. AIA was selected as the marker for its high stability in the gastrointestinal tract of dairy cows, complete resistance to rumen microbial degradation and exogenous enzyme interference, and stable recovery rate (>95%) [27]. The AIA content in feed and fecal samples was determined according to the AOAC 942.05 method, consistent with the procedure described by Furuichi and Takahashi [27]. Apparent total tract digestibility of each nutrient was calculated from the concentrations of AIA (marker) and target nutrient in the feed and feces (all expressed as % in DM) according to the following formula:


(2) Apparent digestibility (%)=100-([Marker in feed×Nutrient in feces]/[Marker in feces×Nutrient in feed])×100[
27].

While collecting urine samples from dairy cows, midstream urine was collected using the vulvar massage stimulation method, to which 10% dilute H_2_SO_4_ was added immediately before storage at 4°C. Urine samples collected over three consecutive days were mixed in a 1:1:1 volume ratio and stored at −20°C. Subsequently, the total N content in the urine samples was measured using a Kjeltec8400 analyzer (Foss). Urea N, creatinine (CREA), and uric acid in urine were determined using commercial kits (Nanjing Jiancheng Bioengineering), while allantoin was analyzed by high-performance liquid chromatography as proposed by Chen and Gomes [26]. According to the method proposed by Valadares et al. [28] daily urine volume of dairy cows was estimated based on CREA content, with the corresponding formula: Urine volume (L/d) = (Body weight [kg]×29 mg/kg)/Urinary creatinine concentration (mg/L). Total purine derivatives (PD) content was calculated as total urine volume×PD concentration, and microbial nitrogen (MN) synthesis was determined according to the method recommended by Chen and Gomes [26].

### Statistical analysis

All data were analyzed using the MIXED procedure in SAS (ver. 9.4; SAS Institute). For parameters measured over time (DMI, milk yield, and milk composition), a repeated measures analysis was performed. The model included the fixed effects of dietary treatment, time, and their interaction, as well as the random effect of cow within group. To account for baseline differences at the start of the experiment (day 0), measurements taken on day 0 were included as a covariate in the model. For parameters measured at a single time point,the model included the fixed effect of treatment and the random effect of cow within group. The overall significance of the treatment effect was determined using an F-test (p<0.05). Results are presented as means±standard errors of the mean (SEM). For parameters with a significant overall treatment effect that were relevant for group-wise comparisons, post-hoc pairwise comparisons were performed using the Tukey-Kramer adjustment. Differences were considered statistically significant at p<0.05, and a tendency toward significance was indicated by 0.05≤p<0.10.

## RESULTS

### Complex enzyme preparations supplementation in low-protein amino acid-balanced diet improves production performance and milk composition of mid-lactation dairy cows

As shown in Table 2, no significant differences were observed in DMI among the treatment groups (p>0.05). Milk yield was significantly affected by dietary treatments, with the LPEP group showing significantly higher production than both the NP and LP groups (p = 0.037), while no significant difference was detected between NP and LP groups. A significant treatment×time interaction was observed for milk yield (p = 0.031). Milk component yields followed similar patterns. Lactose yield was significantly higher in the LPEP group compared to both NP and LP groups (p = 0.043). Milk fat yield showed significant differences among all three groups (p = 0.047), with the highest value in the LPEP group, intermediate in the LP group, and lowest in the NP group. Milk protein yield was significantly greater in the LPEP group than in both NP and LP groups (p = 0.045). Time effects and treatment × time interactions were highly significant for all milk component yields (p<0.001). ECM differed significantly among treatments (p = 0.026), with the LPEP group exhibiting the highest value, followed by the LP group, and the NP group. A similar trend was observed for FCM, with all three groups differing significantly from each other (p = 0.017). Feed efficiency varied significantly among treatments, with the LPEP group showing superior efficiency compared to both NP and LP groups (p<0.05), and the LP group exceeding the NP group (p<0.05). Significant treatment×time interactions were also detected for ECM, FCM, and feed efficiency (p<0.05).

### Complex enzyme preparations supplementation in low-protein amino acid-balanced diet increases apparent digestibility of mid-lactation dairy cows

The results presented in Table 3 demonstrate that supplementing a LP, AA-balanced diet with a complex enzyme preparations significantly enhanced the apparent digestibility of nutrients in mid-lactation dairy cows. Specifically, the apparent digestibility of DM was significantly higher in the LPEP group than in both the NP and LP groups (p<0.05), and the LP group also exhibited a significantly higher DM digestibility than the NP group (p<0.05). For NDF, the LPEP group showed significantly greater digestibility than the NP group (p<0.05). No significant differences in NDF digestibility were observed between the LP and NP groups or between the LPEP and LP groups (p>0.05). The apparent digestibility of ADF was significantly elevated in the LPEP group compared to the NP and LP groups, with the LP group also surpassing the NP group (p<0.05). A similar pattern was found for EE, where the LPEP group had significantly higher digestibility than the NP and LP groups (p<0.05). Finally, the apparent digestibility of CP was significantly greater in the LPEP group than in the NP and LP groups (p<0.05), and the LP group also showed a significant increase over the NP group (p<0.05).

### Effects of complex enzyme preparations supplementation in low-protein amino acid-balanced diet on serum biochemical indicators of mid-lactation dairy cows

The results presented in Table 4 indicate that there were no statistically significant differences in the serum concentrations of total protein (TP), β-hydroxybutyrate (BHBA), total cholesterol (TC), and CREA among the groups of cows (p>0.05). The serum albumin (ALB) concentration in the LPEP group was significantly higher than that in both the NP group and the LP group (p<0.05). In contrast, there was no significant difference in serum ALB concentration between the LP group and the NP group (p>0.05). Concurrently, the serum globulin (GLOB) concentration in the LPEP group was significantly lower than in the LP and NP groups (p<0.05), while there was no significant difference in GLOB concentration between the LP and NP groups (p>0.05). The blood urea nitrogen (BUN) concentrations in both the LPEP and LP groups were significantly lower than that in the NP group (p<0.05). However, the BUN concentration in the LPEP group was only numerically lower than that in the LP group, with no statistically significant difference observed between these two groups (p>0.05). Compared with the NP group, the glucose concentrations in both the LPEP and LP groups decreased. Specifically, the glucose concentration in the LPEP group was significantly lower than that in the NP group (p<0.05), while there was no significant difference in glucose concentration between the LP group and the NP group (p>0.05).

### Effects of complex enzyme preparations supplementation in low-protein amino acid-balanced diet on plasma amino acid composition of mid-lactation dairy cows

The results presented in Table 5 indicate that the plasma total EAA content in the LPEP and LP groups was numerically greater than that in the NP group, and the plasma total EAA content in the LPEP group was numerically higher than in the LP group, but the differences among groups were not statistically significant (p>0.05). Concurrently, compared to the NP group, the plasma concentrations of Lys and Met in both the LPEP and LP groups were significantly elevated (p<0.05), with no significant differences between the LP and LPEP groups (p>0.05). Furthermore, there were no statistically significant differences among the remaining EAA groups (p>0.05). In the NEAA group, only the Aspartate (Asp) concentration in the LPEP group was significantly higher than in the NP group (p<0.05). The differences in other NEAA concentrations among the groups were not statistically significant (p>0.05).

### Effects of complex enzyme preparations supplementation in low-protein amino acid-balanced diet on milk amino acid composition of mid-lactation dairy cows

The results from Table 6 show that there are no statistically significant differences in the Total EAA content and the content of individual EAAs among the groups of milk (p>0.05). Additionally, except for Glycine (Gly), which is significantly higher in the LPEP group compared to the NP and LP group (p<0.01), while the NEAA content in the milk of the other groups shows no statistical differences (p>0.05). It is noteworthy that in the milk of the LP group, Lys, Met, and Total EAA are numerically higher than in the NP and LPEP groups, but these differences are not statistically significant (p>0.05).

### Complex enzyme preparations supplementation in low-protein amino acid-balanced diet improves rumen fermentation parameters of mid-lactation dairy cows

The impact of enzyme preparation in a LP, AA-balanced diet on ruminal fermentation parameters in mid-lactation dairy cows is presented in Table 7. No significant differences were observed in ruminal pH among the treatment groups (p> 0.05). The concentration of NH_3_-N was significantly lower in the LPEP group than in both the NP and LP groups (p<0.05), and the LP group also exhibited a significantly lower NH_3_-N concentration than the NP group (p<0.05). The content of MCP was significantly higher in the LPEP group than in the NP and LP groups (p<0.05), with the LP group also showing a significant increase over the NP group (p<0.05). The acetate concentration was significantly elevated in the LPEP group compared to the NP and LP groups (p<0.05), and the LP group also had a higher acetate concentration than the NP group (p<0.05). The LPEP group demonstrated a significantly higher propionate content than the NP group (p<0.05). No significant differences were detected among the groups for the concentrations of butyrate, valerate, isobutyrate, or isovalerate (p>0.05). The TVFA content was significantly greater in the LPEP group than in the NP and LP groups (p<0.05), and the LP group also registered a significant increase over the NP group (p<0.05). Furthermore, the acetate-to-propionate ratio (A/P) was significantly lower in the LPEP group than in the NP group (p<0.05).

### Complex enzyme preparations supplementation in low-protein amino acid-balanced diet enhances nitrogen utilization efficiency and nitrogen balance in dairy cows

The effects of enzyme preparation in a LP, AA-balanced diet on N utilization efficiency and N balance in mid-lactation dairy cows are presented in Table 8. N intake was significantly lower in both the LPEP and LP groups compared to the NP group (p<0.05), with no significant difference observed between the LPEP and LP groups (p>0.05). The Milk N content was significantly higher in the LPEP group than in both the LP and NP groups (p<0.05). Fecal N output was significantly reduced in the LP and LPEP groups relative to the NP group (p<0.05). The LPEP group exhibited a significantly lower Urine N output than both the NP and LP groups, and the LP group also had significantly lower Urine N than the NP group (p<0.01). Total excreted N was significantly lower in the LP and LPEP groups than in the NP group (p<0.05). N secretion and excretion were significantly reduced in the LPEP and LP groups compared to the NP group (p<0.05), but no significant difference was found between the two LP groups. Both the LPEP and LP groups demonstrated significantly higher N utilization efficiency than the NP group (p<0.05), with the LPEP group being superior to the LP group (p<0.05). Finally, N balance was significantly improved in the LPEP group compared to both the NP and LP groups (p<0.05), and the LP group also showed a significantly higher N balance than the NP group (p<0.05).

## DISCUSSION

This study systematically evaluated the effects of supplementing a LP, AA-balanced diet with a composite enzyme preparations on production performance, nutrient digestibility, N metabolism, and ruminal fermentation in lactating dairy cows. It revealed a synergistic mechanism between the complex enzyme preparations and RP-AA for optimizing N utilization and sustaining high production performance. The results demonstrate that the combined use of RP-Lys, RP-Met, and enzymes in a LPD not only significantly enhanced milk yield, milk composition, and feed efficiency but also further improved apparent nutrient digestibility, optimized ruminal fermentation patterns, and promoted N balance, while effectively reducing N excretion. These findings provide a crucial theoretical and practical foundation for a nutritional strategy in LPD systems that focuses on both enhancing output while reducing environmental impact.

The significant increases in milk yield, ECM, and 4% FCM observed in the LPEP group are closely associated with the dual optimization of nutrient supply and ruminal function achieved through the combined action of the complex enzyme preparations and RP-AAs. On one hand, the composite enzyme preparations containing cellulase, xylanase, and other components effectively degrades non-starch polysaccharide (NSP) structures in roughage, disrupting the physical barriers formed by cellulose and hemicellulose in plant cell walls [29, 30]. This degradation enhances the release of intracellular nutrients such as starch and proteins, thereby providing additional fermentable substrates for rumen microorganisms [17,31]. The elevated apparent digestibility of DM in the LPEP group compared to the LP group directly confirms that enzyme preparation improves the utilization efficiency of the basal diet. On the other hand, the RP-Lys and RP-Met supplemented in the LPD bypass ruminal degradation and are directly absorbed in the small intestine, compensating for the deficiency of limiting AAs and supplying adequate precursors for milk protein synthesis. Notably, the LP group also demonstrated significantly higher milk fat content and ECM yield than the NP group, which aligns with the theory that “amino acid balance reduces protein catabolism”. In high-protein diets, excess AAs are deaminated for energy utilization or urea synthesis, consuming metabolic energy that could otherwise be directed toward milk synthesis [32,33]. In contrast, the balanced AA profile in the LPD allowed for more efficient utilization of absorbed AAs for milk protein synthesis. Meanwhile, compared with the LPEP group, the LP group without enzyme preparation showed lower fiber digestion and subsequent lower energy availability, which was consistent with the significant difference in milk yield between these two groups [34–37]. Furthermore, this synergistic improvement in production performance may involve deeper metabolic regulation [38,39]. Related studies indicate that enzyme preparation enhances ruminal fermentation in dairy cows, leading to increased production of VFAs, particularly propionate [38]. This increase may provide additional precursors for hepatic gluconeogenesis, thereby optimizing the body’s energy metabolism status Leão et al. [39]. Consequently, the incorporation of the complex enzyme preparations essentially achieves a breakthrough in production performance through dual pathways: enhancing nutrient digestibility and optimizing metabolic partitioning.

Before elucidating the promotional effect of complex enzyme preparations on nutrient digestibility, we first clarified the underlying mechanism of digestibility differences between the LP and NP groups, to address the differential response of fiber fractions to the LP AA-balanced diet. In the present study, compared with the NP group, the LP group showed a significant elevation in the apparent total tract digestibility of DM and ADF, with no significant alteration in NDF digestibility. Notably, this effect was not induced by the direct modulation of ruminal digestion via RP-AAs. The primary biological role of RP-AAs is to replenish limiting AAs in the small intestine post-ruminal bypass, thus enabling the successful implementation of the LPD strategy, rather than directly intervening in ruminal degradation processes. The improved DM digestibility observed in the LP group is a combined consequence of optimized dietary formulation and enhanced rumen microecological homeostasis, which aligns with previous findings reported by Seleem et al. [14] and Gunun et al. [33]. The differential digestibility response between NDF and ADF is fundamentally attributed to their inherent structural discrepancies [20]: NDF is composed of cellulose, hemicellulose and lignin, with its degradation constrained by multiple structural barriers, while ADF is predominantly cellulose-based, which acts as the preferential degradation substrate for ruminal fibrolytic bacteria. Coupled with the optimization of fiber ingredients in the LP diet, this ultimately resulted in the observed digestibility pattern, which is consistent with the conclusions of a homologous study conducted by Erickson et al. [9].

The supplementation with the complex enzyme preparations significantly enhanced the apparent digestibility of DM, NDF, ADF, and CP in lactating dairy cows. This improvement is likely attributable to the effective degradation of structural carbohydrates in the feed by non-starch polysaccharidases, such as cellulase and xylanase, which liberates additional available carbon sources and thereby supplies ample energy for the growth and metabolism of ruminal microbiota [40–42]. The present study observed a significant decrease in ruminal NH_3_-N concentration alongside a marked increase in MCP synthesis in the LPEP group. This indicates that the complex enzyme preparations promoted the capture and utilization of N sources by rumen microbes via enhanced fiber degradation, consequently reducing ammonia loss and improving MCP synthesis efficiency [43,44]. This mechanistic shift is consistent with the significant reduction in BUN concentration, providing further evidence of a transition towards more efficient N metabolism. Furthermore, the elevated apparent digestibility in the LPEP group may also reflect the multi-target action of the complex enzyme preparations in counteracting anti-nutritional factors. The cellulases and xylanases specifically hydrolyze β-1,4-glycosidic bonds in cellulose and hemicellulose, reducing NDF crystallinity and increasing the surface area available for microbial colonization [45,46]. This effect is directly corroborated by the higher NDF and ADF digestibility in the LPEP group compared to the LP group.

Serum ALB, a positive indicator of protein nutritional status and hepatic synthesis function, was significantly elevated in the LPEP group, suggesting superior protein nutrition and a healthier metabolic state in these cows. Although Zhang et al. [47] highlighted the link between liver function and protein metabolism in transition cows, the elevated ALB observed in our mid-lactation cows similarly reflects enhanced hepatic synthetic capacity, likely mobilized to support the demands of high milk yield. Concurrently, the significantly reduced BUN concentration provides direct circulatory evidence of improved dietary N utilization. This finding aligns with the lower ruminal NH_3_-N concentration observed, collectively depicting a more efficient N metabolism pathway from the rumen to the systemic circulation. This correlation is consistent with the findings of Ferreira et al. [48], who also reported a declining trend in BUN following enzyme preparation. Furthermore, the moderate decreases in GLOB and blood glucose levels suggest a more stable metabolic status, potentially attributable to improved energy-N balance and a reduced hepatic metabolic load [49,50]. Notably, we found that the glucose concentration in the LPEP group was significantly lower than that in the NP group. We speculate that this may be attributed to the supplementation of RP-AAs, which enhances insulin sensitivity in peripheral tissues and promotes greater glucose uptake and utilization for milk synthesis and body tissue growth. In addition, the increased ruminal propionate production enhanced hepatic gluconeogenesis and reduced circulating glucose mobilization, while the elevated milk and lactose yield increased mammary glucose uptake for lactose synthesis, and these factors were important causes of this change. This explanation is consistent with the increased milk yield and milk component yield observed in the LPEP group (Table 2). To clarify the mechanisms underlying this result, we will further determine the relevant indicators, including insulin sensitivity, hepatic glucose output, and mammary glucose uptake, in subsequent studies.

It is noteworthy that the plasma AA profile in this study revealed significantly higher concentrations of Lys and Met in the LPEP and LP groups compared to the NP group, confirming the efficacy of RP-AA supplementation. Asp, an intermediate in the tricarboxylic acid (TCA) cycle, participates in the synthesis of other AAs and energy metabolism. Increased concentration of this metabolite was observed in the LPEP group, which may be associated with the improved nutrient digestion and rumen fermentation profile induced by complex enzyme preparations. However, the milk AA profile did not show corresponding significant alterations, with only Gly being significantly elevated in the LPEP group. Gly is an important component of milk casein and a precursor for glutathione [51]. Its increased concentration may be associated with improved energy supply promoting the conversion of Ser to Gly, or enhanced protein digestion increasing dietary Gly absorption. The lack of synchronized changes in the milk AA profile may be related to the complexity of AA uptake, metabolism, and the regulation of milk protein synthesis in the mammary gland [52,53]. These results suggest that although dietary AA supply was improved, its conversion efficiency into milk protein remains influenced by multifactorial regulation.

The rumen is the central organ for nutritional regulation in ruminants, and our study yielded pivotal findings at this site. The LPEP group exhibited lower ruminal NH_3_-N concentration alongside increased MCP synthesis and TVFA production. This combination of “one decrease and two increases” constitutes a gold-standard indicator of improved energy-N synchrony in the rumen. Related studies have indicated that supplementing diets with composite enzyme preparations can significantly alter the concentrations of TVFA and acetate, while substantially increasing the degradation rates of CP and NDF [54]. Their results are closely aligned with our findings. This synchrony enables the efficient conversion of N that might otherwise be excreted as urea into MCP. This process not only enhances NUE but also ensures a steady supply of high-quality MCP to the small intestine, thereby meeting the host’s AA requirements and establishing a virtuous nutritional cycle. Furthermore, the concurrent increase in acetate and propionate concentrations, coupled with a reduced A/P in the LPEP group, indicates a significant shift in rumen fermentation patterns. A higher propionate proportion generally indicates greater energy efficiency, as propionate serves as the primary gluconeogenic precursor in ruminants. This aligns with the conclusions of Pech-Cervantes et al. [54] in a meta-analysis, which found that exogenous fibrolytic enzymes consistently increased the propionate proportion in the rumen. This optimized fermentation pattern completes the logical sequence connecting it to the observed production performance: improved ruminal fermentation supplies more and better-balanced nutrients, ultimately supporting greater milk production efficiency. This also suggests that enzyme preparation not only promotes fiber fermentation but may also optimize the utilization of non-fibrous components like starch, thereby enhancing the overall energy supply efficiency of the rumen [55–57].

N metabolism and balance constituted another central focus of this investigation. Fecal N, primarily derived from undigested dietary protein and microbial residues, was lower in the LPEP group than in the NP group, this reduction was a direct consequence of the LPEP group’s higher CP digestibility, which decreased the excretion of undigested protein. Urinary N, predominantly in the form of urea, was also reduced in the LPEP group. This reduction may be attributed to decreased BUN concentration reducing renal urea filtration, and/or to improved ruminal fermentation and MCP synthesis, which enhanced the flow and balance of AAs to the small intestine. The AA profile of MCP is closer to that of milk protein, granting it a higher biological value than some rumen-bypass proteins [58,59]. Consequently, the mammary gland can utilize the absorbed AAs more efficiently for milk protein synthesis, reducing catabolism and urinary N excretion caused by AA imbalances. Hernandez [60] emphasized that meeting the specific AA requirements of the mammary gland is crucial for enhancing milk protein synthesis. Our findings further reveal that optimizing ruminal function vian a complex enzyme preparations is an efficient and cost-effective strategy to achieve this goal.

Compared with previous studies, our findings are consistent with some reports on LPD or enzyme supplements used individually, but they also demonstrate distinct synergistic benefits. RP-AAs compensate for limiting AA deficiency caused by dietary protein reduction, maintain lactation performance and stable rumen microbial environment, and lay a necessary foundation for the enzyme preparation to function; the complex enzyme preparations breaks through the core bottlenecks of LPD by degrading plant cell walls, enhancing ruminal fermentable energy supply, and achieving rumen energy-N synchrony, which amplifies the beneficial effects of RP-AAs. In summary, the synergistic effect of the two additives fully verifies the core scientific hypothesis proposed in the Introduction, and provides a theoretical basis for the combined application of enzyme preparation and RP-AAs in LP dairy rations.

## CONCLUSION

In summary, this study provides the first systematic evaluation of the comprehensive effects of enzyme preparation in LP, AA-balanced diets for lactating dairy cows. It clearly demonstrates the synergistic effects of this approach in enhancing production performance, optimizing ruminal fermentation, and improving N utilization, thereby offering a novel perspective for developing efficient and environmentally friendly dairy rations. These findings provide valuable insights for reducing reliance on dietary protein in precision dairy nutrition and contribute to the development of strategies for sustainable livestock production.

## Figures and Tables

**Table 1:** Ingredients of experimental diets (ingredients and composition, % of DM unless otherwise noted) fed to cows

Item	Treatment^1)^		

	NP	LP	LPEP
Whole cottonseed	2.00	2.00	2.00
Flaked corn	0.85	5.45	5.45
Alfalfa (RFV150)	4.20	2.80	2.80
Corn silage	42.50	37.00	37.00
Sugar beet pellets	-	2.30	2.30
Baking soda	1.10	1.90	1.90
Calcium fatty acids	1.15	1.30	1.30
Cornmeal fines	19.70	13.70	13.70
Soybean meal	13.50	19.51	19.51
Inflated soybeans	1.50	5.50	5.50
Premix^2)^	2.50	4.00	4.00
Distillers dried grains with solubles (DDGS)	5.00	-	-
Canola meal	2.00	4.00	4.00
Cottonseed meal CP 46%	2.00	-	-
Wheat bran	2.00	-	-
Rumen-protected methionine (RP-Met)^3)^	-	0.20	0.20
Rumen-protected lysine (RP-Lys)^4)^	-	0.14	0.14
Urea (CP200%)	-	0.20	0.20
Complex enzyme preparations^5)^	-	-	20
Nutrient composition (% DM)^6)^			
DM (%)	88.50	88.20	88.20
CP (%)	17.30	16.09	16.09
EE (%)	4.19	3.90	3.90
Starch (%)	27.79	29.39	29.39
NDF (%)	27.35	27.75	27.75
ADF (%)	17.30	17.23	17.23
Ca (%)	0.83	0.81	0.81
P (%)	0.43	0.35	0.35
Lys (%MP)^7)^	6.43	6.82	6.82
Met (%MP)^7)^	1.88	2.22	2.22
NEL (Mcal/kg)	1.71	1.72	1.72

1) NP: normal protein diet (17.30% CP), no supplementation of RP-AA or complex enzyme preparations; LP: low-protein amino acid-balanced diet (16.09% CP)+RP-Lys+RP-Met; LPEP: LP group diet+20 g/head/d complex enzyme preparations.

2) Each kg of premix contains: VA 7,360 IU, VD 850 IU, VE 1.84 IU, Zn 0.21 mg, Co 0.002 mg, I 0.002 mg, Mn 0.15 mg, Se 0.001 mg, Cu 0.03 mg; The premix content differs between NP and LP/LPEP groups because the RP-AAs (RP-Met and RP-Lys) were incorporated into the premix prior to TMR mixing. The increased premix amount in LP and LPEP groups (4.00% vs. 2.50% in NP) serves as a carrier to ensure uniform distribution of these supplemental amino acids throughout the diet. The premix composition was adjusted accordingly to maintain consistent vitamin and mineral levels across all treatment groups on a total diet basis.

3) LP and LPEP groups were supplemented with 30 g/head/d RP-Met (incorporated into the diet, corresponding to 0.20% of DM basis).

4) LP and LPEP groups were supplemented with 20 g/head/d RP-Lys (incorporated into the diet, corresponding to 0.14% of DM basis).

5) Complex enzyme preparations SFC-061 (Cangzhou Xiasheng Enzyme Biotechnology), with enzymatic activities: cellulase≥1,600 U/g, xylanase≥11,200 U/g, β-glucanase≥3,000 U/g, neutral protease≥500 U/g, β-mannase≥30 U/g; moisture≤8%. The complex enzyme preparations was added at 20 g/head/d.

6) The content of main nutrients is based on DM, net energy is calculated value, and other nutrient levels were measured.

7) %MP: percentage of amino acid in metabolizable protein, predicted based on NRC (2001) model and verified by dietary amino acid analysis.

DM, dry matter; CP, crude protein; EE, ether extract; NDF, neutral detergent fiber; ADF, acid detergent fiber; Ca, calcium; P, phosphorus; Lys, lysine; MP, metabolizable protein; Met, methionine; NEL, net energy for lactation; RP-AA, rumen-protected amino acid; TMR, total mixed ration.

**Table 2:** Effects of enzyme preparation in a low-protein, amino acid-balanced diet on the performance of mid-lactation dairy cows (n = 30)

Item	Group			SEM	p-value		
	
	NP	LP	LPEP		Trt	Time	Trt×Time
DMI (kg/d)	24.80	24.22	24.40	0.142	0.996	0.742	0.464
Milk yield (kg/d)	35.39^b^	35.83^b^	37.84^a^	0.236	0.037	0.092	0.031
Milk component yield							
Lactose (kg/d)	1.82^b^	1.85^b^	1.96^a^	0.071	0.043	0.014	<0.001
Fat (kg/d)	1.32^c^	1.49^b^	1.55^a^	0.112	0.047	0.012	<0.001
Protein (kg/d)	1.26^b^	1.26^b^	1.34^a^	0.061	0.045	0.032	<0.001
ECM (kg/d)^1)^	33.92^c^	34.77^b^	36.33^a^	0.115	0.026	0.037	0.027
FCM (kg/d)^2)^	33.37^c^	34.43^b^	36.40^a^	0.133	0.017	0.044	0.029
Feed efficiency^3)^	1.43^c^	1.48^b^	1.55^a^	0.011	0.039	0.029	<0.001

1) ECM (kg/d) = 0.327×milk yield (kg/d)+12.95×milk fat yield (kg/d)+7.65×milk protein yield (kg/d).

2) FCM (kg/d) = 0.4×milk yield (kg/d)+15×milk fat yield (kg/d).

3) Feed efficiency = ECM (kg)/DMI (kg).

a–c Means within a row with different superscripts differ (p<0.05).

NP, normal protein; LP, low-protein; LPEP, LP group diet+20 g/head/d complex enzyme preparations; SEM, standard errors of the mean; DMI, dry matter lntake; ECM, energy corrected milk; FCM, fat corrected milk.

**Table 3:** Effects of enzyme preparation in a low-protein, amino acid-balanced diet on apparent digestibility in mid-lactation dairy cows (n = 30)

Apparent digestibility (%)	Group			SEM	p-value

	NP	LP	LPEP		
DM	62.53^c^	65.18^b^	69.92^a^	1.032	0.034
NDF	63.18^b^	65.56^ab^	67.85^a^	0.790	0.047
ADF	51.35^c^	57.63^b^	62.34^a^	1.151	0.038
EE	69.65^b^	73.61^ab^	75.28^a^	3.314	0.042
CP	61.25^c^	65.38^b^	69.61^a^	1.371	0.039

a–c Means within a row with different superscripts differ (p<0.05).

NP, normal protein; LP, low-protein; LPEP, LP group diet+20 g/head/d complex enzyme preparations; SEM, standard errors of the mean; DM, dry matter; NDF, neutral detergent fiber; ADF, acid detergent fiber; EE, ether extract; CP, crude protein.

**Table 4:** Effects of enzyme preparation in a low-protein, amino acid-balanced diet on serum biochemical parameters in mid-lactation dairy cows (n = 30)

Item	Group			SEM	p-value

	NP	LP	LPEP		
TP (g/L)	68.98	71.79	70.45	2.572	0.745
ALB (g/L)	33.78^b^	37.27^b^	42.78^a^	2.344	0.041
GLOB (g/L)	35.50^a^	34.33^a^	27.67^b^	4.335	0.032
BUN (mmol/L)	5.05^a^	3.58^b^	3.03^b^	0.333	0.016
BHBA (mmol/L)	0.26	0.20	0.23	0.071	0.810
TC (mmol/L)	7.41	6.20	5.72	0.854	0.454
Glu (mmol/L)	3.22^a^	2.79^ab^	2.42^b^	0.286	0.039
CREA (μmol/L)	52.53	62.47	61.42	1.062	0.392

a,b Means within a row with different superscripts differ (p<0.05).

NP, normal protein; LP, low-protein; LPEP, LP group diet+20 g/head/d complex enzyme preparations; SEM, standard errors of the mean; TP, total protein; ALB, albumin; GLOB, globulin; BUN, blood urea nitrogen; BHBA, beta-hydroxybutyric acid; TC, total cholesterol; Glu, glucose; CREA, creatinine.

**Table 5:** Effects of enzyme preparation in a low-protein, amino acid-balanced diet on plasma amino acid profile in mid-lactation dairy cows (n = 30)

Item^1)^	Group			SEM	p-value

	NP	LP	LPEP		
EAA (μg/mL)					
Arg (μg/mL)	8.85	10.03	9.42	1.662	0.881
His (μg/mL)	11.20	11.40	12.10	1.101	0.837
Ile (μg/mL)	17.12	18.72	20.35	2.962	0.751
Leu (μg/mL)	22.46	23.99	25.95	4.254	0.845
Lys (μg/mL)	6.80^b^	11.22^a^	10.81^a^	1.211	<0.001
Met (μg/mL)	2.43^b^	3.23^a^	3.33^a^	0.254	0.039
Phe (μg/mL)	6.81	8.98	9.31	1.051	0.399
Thr (μg/mL)	7.89	9.00	10.47	1.203	0.435
Val (μg/mL)	31.72	30.04	31.59	5.942	0.835
Total EAA (μg/mL)	132.10	137.80	144.50	19.014	0.868
Total EAA-Lys-Met (μg/mL)	122.10	124.70	131.70	16.677	0.725
NEAA					
Ala (μg/mL)	24.21	22.14	23.35	3.083	0.634
Asp (μg/mL)	0.95^b^	1.49^ab^	1.85^a^	0.246	0.042
Glu (μg/mL)	7.14	10.05	9.19	1.392	0.141
Gly (μg/mL)	19.71	20.43	24.48	3.74	0.517
Pro (μg/mL)	10.96	9.33	10.71	1.113	0.549
Ser (μg/mL)	29.08	25.13	22.69	2.531	0.342
Tyr (μg/mL)	9.55	9.22	10.72	1.519	0.579
Total NEAA (μg/mL)	101.6	97.78	103.00	7.667	0.853

1) All plasma amino acids measured in this study refer to free amino acids (after protein precipitation).

a,b Means within a row with different superscripts differ (p<0.05).

NP, normal protein; LP, low-protein; LPEP, LP group diet+20 g/head/d complex enzyme preparations; SEM, standard errors of the mean; EAA, essential amino acid; Arg, arginine; His, histidine; Ile, isoleucine; Leu, leucine; Lys, lysine; Met, methionine; Phe, phenylalanine; Thr, threonine; Val, valine; NEAA, non-essential amino acid; Ala, alanine; Asp, aspartic acid; Glu, glutamic acid; Gly, glycine; Pro, proline; Ser, serine; Tyr, tyrosine.

**Table 6:** Enzyme preparation in a low-protein, amino acid-balanced diet modifies the milk amino acid profile in mid-lactation dairy cows (n = 30)

Item	Group			SEM	p-value

	NP	LP	LPEP		
EAA (μg/mL)					
Arg (μg/mL)	3.28	2.84	2.95	0.524	0.833
His (μg/mL)	1.46	1.35	1.41	0.112	0.619
Ile (μg/mL)	4.6	4.16	4.65	0.353	0.511
Leu (μg/mL)	1.5	1.2	1.26	0.284	0.572
Lys (μg/mL)	4.08	6.77	3.40	2.176	0.443
Met (μg/mL)	0.19	0.39	0.25	0.129	0.565
Phe (μg/mL)	0.32	0.32	0.36	0.071	0.889
Thr (μg/mL)	0.99	0.77	1.11	0.252	0.661
Val (μg/mL)	3.21	2.25	3.39	1.075	0.733
Total EAA (μg/mL)	19.73	20.16	18.88	3.094	0.955
Total EAA-Lys-Met (μg/mL)	15.46	13.00	15.24	1.927	0.636
NEAA					
Ala (μg/mL)	3.02	2.64	4.42	0.942	0.594
Asp (μg/mL)	1.41	0.94	1.94	0.471	0.922
Glu (μg/mL)	40.30	20.64	51.85	14.371	0.446
Gly (μg/mL)	1.59^b^	1.89^b^	6.29^a^	1.412	<0.001
Pro (μg/mL)	2.81	2.45	2.92	0.615	0.851
Ser (μg/mL)	28.81	25.63	25.68	1.223	0.430
Tyr (μg/mL)	0.57	0.5	0.57	0.146	0.715
Total NEAA (μg/mL)	79.06	55.97	94.38	17.134	0.464

a,b Means within a row with different superscripts differ (p<0.05).

NP, normal protein; LP, low-protein; LPEP, LP group diet+20 g/head/d complex enzyme preparations; SEM, standard errors of the mean; EAA, essential amino acid; Arg, arginine; His, histidine; Ile, isoleucine; Leu, leucine; Lys, lysine; Met, methionine; Phe, phenylalanine; Thr, threonine; Val, valine; NEAA, non-essential amino acid; Ala, alanine; Asp, aspartic acid; Glu, glutamic acid; Gly, glycine; Pro, proline; Ser, serine; Tyr, tyrosine.

**Table 7:** Effects of enzyme preparation in a low-protein, amino acid-balanced diet on rumen fermentation parameters in mid-lactation dairy cows (n = 30)

Item	Group			SEM	p-value

	NP	LP	LPEP		
pH	6.46	6.39	6.38	0.092	0.768
NH_3_-N (mg/dL)	12.24^a^	10.16^b^	7.30^c^	0.804	0.026
MCP (mg/dL)	1.68^c^	1.76^b^	1.85^a^	0.041	0.029
Acetate (mmol/L)	62.61^c^	66.13^b^	69.60^a^	1.854	0.045
Propionate (mmol/L)	23.35^b^	25.32^ab^	27.86^a^	1.447	0.025
Butyrate (mmol/L)	10.16	10.81	11.61	0.652	0.725
Valerate (mmol/L)	1.42	1.47	1.50	0.02	0.867
Isobutyrate (mmol/L)	0.82	0.83	0.86	0.022	0.855
Isovalerate (mmol/L)	1.53	1.56	1.54	0.014	0.787
TVFA (mmol/L)	99.89^c^	106.12^b^	112.97^a^	2.096	0.025
Acetate: Propionate	2.68^a^	2.61^ab^	2.50^b^	0.125	0.042

a–c Means within a row with different superscripts differ (p<0.05).

NP, normal protein; LP, low-protein; LPEP, LP group diet+20 g/head/d complex enzyme preparations; SEM, standard errors of the mean; pH, potential of hydrogen; NH_3_-N, ammonia nitrogen; MCP, microbial crude protein; TVFA, total volatile fatty acid.

**Table 8:** Effects of enzyme preparation in a low-protein, amino acid-balanced diet on nitrogen (N) utilization and balance in mid-lactation dairy cows (n = 30)

Item	Group			SEM	p-value

	NP	LP	LPEP		
N intake (g/d)^1)^	686.33^a^	623.52^b^	628.02^b^	10.242	0.018
Milk N (g/d)	201.60^b^	201.60^b^	214.40^a^	0.612	0.025
Fecal N (g/d)	217.06^a^	190.47^b^	184.61^b^	1.194	0.019
Urine N (g/d)	175.36^a^	135.25^b^	128.46^c^	1.463	0.002
Total excreta N (g/d)^2)^	392.42^a^	325.72^b^	313.07^b^	1.152	0.037
N secretion and excretion (g/d)^3)^	594.02^a^	527.32^b^	527.47^b^	0.251	0.039
N utilization efficiency (%)^4)^	29.37^c^	32.33^b^	34.14^a^	0.181	0.030
N balance (g/d)^5)^	92.31^c^	96.20^b^	100.55^a^	0.168	0.034

1) N intake = 1,000×DMI (g)×Dietary protein level (17.30%/16.09%)×0.16.

2) Total excreta N = Fecal N (g/d)+Urine N (g/d).

3) N secretion and excretion = Milk N (g/d)+Fecal N (g/d)+Urine N (g/d).

4) Nitrogen utilization efficiency = 100×Milk protein N (g/d)/N intake (g/d).

5) N balance (g/d) = Intake N−(Milk N+Fecal N+Urine N) (g/d).

a–c Means within a row with different superscripts differ (p<0.05).

NP, normal protein; LP, low-protein; LPEP, LP group diet+20 g/head/d complex enzyme preparations; SEM, standard errors of the mean.

## Data Availability

Upon reasonable request, the datasets of this study can be available from the corresponding author.
